# Oxidative Stability and Protective Effect of the Mixture between *Helianthus annuus* L. and *Oenothera biennis* L. Oils on 3D Tissue Models of Skin Irritation and Phototoxicity

**DOI:** 10.3390/plants11212977

**Published:** 2022-11-04

**Authors:** Ramona Fecker, Ioana Zinuca Magyari-Pavel, Ileana Cocan, Ersilia Alexa, Iuliana Maria Popescu, Adelina Lombrea, Larisa Bora, Cristina Adriana Dehelean, Valentina Buda, Roxana Folescu, Corina Danciu

**Affiliations:** 1 Department of Pharmacognosy, Faculty of Pharmacy, “Victor Babes” University of Medicine and Pharmacy Timisoara, Eftimie Murgu Square No. 2, 00041 Timişoara, Romania; 2 Research Center for Pharmaco-Toxicological Evaluations, Faculty of Pharmacy, “Victor Babes” University of Medicine and Pharmacy Timisoara, Eftimie Murgu Square No. 2, 300041 Timişoara, Romania; 3 Faculty of Food Engineering, University of Life Sciences “King Michael I” from Timisoara, Calea Aradului 119, 300645 Timisoara, Romania; 4 Faculty of Agriculture, University of Life Sciences “King Michael I” from Timisoara, Calea Aradului 119, 300645 Timisoara, Romania; 5 Department of Toxicology, Faculty of Pharmacy, “Victor Babes” University of Medicine and Pharmacy Timisoara, Eftimie Murgu Square No. 2, 300041 Timişoara, Romania; 6 Department of Clinical Pharmacy, Communication in Pharmacy and Pharmaceutical Care, Faculty of Pharmacy, “Victor Babes” University of Medicine and Pharmacy Timisoara, Eftimie Murgu Square No. 2, 300041 Timişoara, Romania; 7 Department of Balneology, Medical Recovery and Rheumatology, Family Discipline, Center for Preventive Medicine, Center for Advanced Research in Cardiovascular Pathology and Hemostaseology, “Victor Babes” University of Medicine and Pharmacy Timisoara, Eftimie Murgu Square No. 2, 300041 Timişoara, Romania

**Keywords:** *Oenothera biennis* L. oil, *Helianthus annuus* L. oil, skin irritation, phototoxicity, 3D tissue models

## Abstract

The present study was aimed to evaluate the oxidative stability as well as to assess the protective effect of the mixture of *Helianthus annuus* L. (HAO) and *Oenothera biennis* L. (OBO) oils on 3D tissue models of skin irritation and phototoxicity. The following methods were used: GS analysis (fatty acids composition), thiobarbituric acid-reactive substances assay (TBA) (lipid oxidation degree of tested samples), 3D EpiDerm models (skin irritation and phototoxicity). For HAO the detected saturated fatty acids (SFA) were palmitic acid (7.179%), stearic acid (3.586%), eicosanoic (0.138%) and docosanoic acid (0.548%) The monounsaturated acids (MUFA) were palmitoleic acid (0.158%) and oleic acid (28.249%) and the polyunsaturated acids (PUFA) were linoleic acid (59.941%) and linolenic acid (0.208%). For OBO the detected SFA were myristic acid (0.325%), pentadecylic acid (0.281%), palmitic (7.2%), stearic (2.88%), and arachidic acid (0.275%). Regarding MUFA, even a lower proportion (8.196%) was observed, predominantly being oleic acid, cis form (7.175%), oleic (n10) (0.558%) and 11-eicosenoic (0.210%) acids. The higher content was found for PUFA (82.247%), the most significant proportions being linoleic acid (72.093%), arachidonic acid (9.812%) and linolenic (0.233%). Obtained data indicate a good oxidative stability and biocompatibility of the mixture on the 3D EpiDerm models with no irritant and no phototoxic effects. *Oenothera biennis* L. oil may be an excellent natural choice in order to delay or prevent oxidative damage of *Helianthus annuus* L. oil.

## 1. Introduction

Plants are valuable resources for the research and development of novel medicines, their usage dateing back to antiquity [1]. Throughout history, a broad selection of oils (both fatty as well as volatile) extracted from different vegetal products have been employed in different branches, including pharmaceuticals, cosmetics and cuisine. Presently, they are widely acknowledged for their benefits in the treatment of a broad range of skin illnesses and other conditions, as well as for their contribution to the preservation and restoration of skin barrier stability [2,3].

The common sunflower (*Helianthus annuus* L.) is a member of the *Asteraceae* family that is cultivated commercially all over the globe and has several dietary and therapeutic properties. In traditional ethnomedicine, sunflower seeds are used to treat bronchial, laryngeal and pulmonary conditions, coughs and colds, and whooping cough [4].

Regarding the chemical composition, it is established that the fatty acids represent the significant and richest part of *Helianthus annuus* L. oil (HAO) alongside a smaller percentage of tocopherols and phytosterols [5]. Furthermore, Spring et al. suggested in a recent study that HAO may also contain bioactive sesquiterpene lactones [6]. Likewise, the composition of fatty acids differs depending on environmental and harvest conditions (irrigation, sowing date, harvest period, soil condition) [7]. Commonly, the standard HAO contains a ratio of 70% polyunsaturated linoleic acid/20% monosaturated oleic acid, representing 90% of the total fatty acid content; palmitic and stearic acids represent 5–15% of fatty acids in the oil [8]. Additionally, sunflower seeds provide substantially more vitamin E (37.8 mg/100 g) than linseed, sesame, and soy (all of which carry only around 3 mg/100 g) [9]. Minerals such as calcium, copper, iron, magnesium, manganese, selenium, phosphorus, potassium, sodium, and zinc are abundant in sunflower seeds [10]. Flavonoids (heliannone, quercetin, kaempferol, luteolin, apigenin) [11], phenolic acids (caffeic acid, chlorogenic acid, caffeoylquinic acid, gallic acid, protocatechuic, coumaric, ferulic acid, and sinapic acid) have been isolated from the sunflower seed and sprout and demonstrated to participate to its medicinal properties [12,13].

Sunflower oil has an important role in the food, cosmetic and pharmaceutical industries [14], as well as a dietary role in nutrition [15]. HAO is known to have anti-inflammatory and antioxidant activities due to the content of vitamin E [16], cardiovascular benefits by lowering the LDL-cholesterol levels and influencing the blood pressure and glucose metabolism [17], protective and healing effects on the skin [18] and antimicrobial activity [14].

*Oenothera biennis* L. (OB), also known as evening primrose, belongs to the *Onagraceae* family [19].

Recent studies have shown that *Oenothera biennis* L. oil (OBO) (obtained from seeds) and OB extract (obtained from aerial parts) are important sources of bioactive compounds with considerable nutritional value. From the chemical point of view OBO contains fatty oils (24%) consisting mainly of linoleic acid (65–80%), *γ*-linoleic acid (8–14%), oleic, palmitic and stearic acid [19]. Withal, it contains important amounts of other fatty acids such as stearic, palmitic, oleo palmitic, myristic, vaccenic, eicosenoic and eicosanoic acids. Additionally, OB represents a rich source of polyphenols, such as hydroxytyrosol (1.11 mg/kg oil), vanillin (17.37 mg/kg oil), vanillic acid (3.27 mg/kg oil), p-coumaric acid (1.75 mg/kg oil), and also ferulic acid (25.23 mg/kg oil) [20]. Moreover, OB seeds contain macroelements (calcium, iron, zinc, magnesium) and also have an essential amino acid profile (tryptophan, lysine, cystin, tyrosine) [21]. 

Previous in vitro studies have revealed that OBO possesses many biological activities such as antioxidant and anti-inflammatory [22], being benefic in women’s health (premenstrual syndrome, mastalgia) [23], rheumatoid arthritis [24] and diabetes [25]. The aerial part of OB extract showed anti-inflammatory [26], antimicrobial [27] and cytotoxic properties [28].

Due to the high content of linoleic, respectively *γ*-linolenic acids, the topical and oral administration of both HAO and OBO improve the skin barrier function by reducing the trans epidermal water loss (TEWL) and by increasing the elasticity and firmness of the skin. In addition, the compounds exert an anti-inflammatory effect on the skin, thus being useful in cutaneous disorders such as atopic dermatitis and acne [16,29].

The oxidative damage caused by the lipid oxidation of sunflower oil can seriously affect human health. Known food additives such as butylate hydroxyanisole (BHA) and tert-butyl hydroquinone (TBHQ) are used in order to delay or prevent oxidative damage. Recent studies show that these synthetic antioxidants may be involved in carcinogenesis. Natural sources of natural antioxidants have been sought lately, with effective inhibitory effects against the oxidative rancidity of sunflower oil [30].

The aim of this study is to evaluate the oxidative stability as well as to assess the protective effect of the mixture between *Helianthus annuus* L. and *Oenothera biennis* L. oils on 3D tissue models of skin irritation and phototoxicity.

## 2. Results

### 2.1. Fatty Acids Composition

The composition of the fatty acids (FA) in the analysed oils sample of *Helianthus annuus* L. (HAO) and Oenothera biennis L. (OBO) is shown in Table 1.

HAO has a low content of saturated fatty acids (SFA), respectively of 11.451%, the identified compounds being palmitic acid (7.179%), stearic acid (3.586%), eicosanoic (0.138%) and docosanoic acid (0.548%). The monounsaturated acids (MUFA) identified are palmitoleic acid (0.158%) and oleic acid (28.249%) and the polyunsaturated fatty acids (PUFA) are linoleic acid in the highest amount (59.941%) and linolenic acid (0.208%). It can also be seen that HAO has a very low content of ω3 fatty acids (0.208%).

As can be seen, OBO has a low rate of saturated fatty acids (SFA), respectively 9.49%, myristic acid being predominant (0.325%), pentadecylic acid (0.281%), palmitic (7.2%), stearic (2.88%), and arachidic (0.275%). Regarding MUFA, even a lower proportion (8.196%) is observed, predominantly being oleic acid, cis form (7.175%), oleic (n10) (0.558%) and 11-eicosenoic (0.210%). The higher content was found for PUFA by 82.247%, the most significant proportions being linoleic acid (72,093%), arachidonic acid (9.812%) and linolenic (0.233%). 

Table 2 shows the fatty acid classes, the ratio of fatty acids and the quality indices, IA and IT.

Regarding the acid classes, the fat content in HAO and OBO was characterized by a higher content of UFA than SFA.

From a nutritional point of view, HAO recorded a significant content of MUFAs (28.407%). At the same time, OBO showed significantly lower values of MUFAs (8.196). Regarding the PUFA content of the two oils, in both cases, high values were recorded, respectively 59.941% for HAO and 72.143% for OBO. For both HAO and OBO, PUFA in the ω6 series recorded significantly higher values than those of the ω3 series. Consequently, the ratio UFA/SFA and PUFA/SFA showed significantly higher values for both oils. In contrast, the PUFA ω3/ω6 ratio was significantly lower. Finally, AI and IT, strictly related to the fatty acid profile, showed significantly reduced values. Regarding the acid classes, the fat content in HAO and OBO was characterized by a higher content of UFA than SFA.

From a nutritional point of view, HAO recorded a significant content of MUFA (28.407%). At the same time, OBO showed significantly lower values of MUFA (8.196%). Regarding the PUFA content of the two oils, in both cases, high values were recorded, respectively 59.941% for HAO and 72.143% for OBO. For both HAO and OBO, PUFA in the ω6 series recorded significantly higher values than those of the ω3 series. Consequently, the ratio UFA/SFA and PUFA/SFA showed significantly higher values for both oils. In contrast, the PUFA ω3/ω6 ratio was considerably lower. Finally, AI and IT, strictly related to the fatty acid profile, showed significantly reduced values. The higher values of PUFA/SFA, UFA/SFA and ω−6 PUFA and the lower values of SFA, TI and AI confirm the higher nutritional value of the lipid fraction in OBO compared to those in HAO. 

### 2.2. Thiobarbituric Acid-Reactive Substances Assay (TBA)

In Table 3 are presented the thiobarbituric acid (TBA) values registered for the tested samples follow the degree of secondary oxidation over a storage period of 30 days at room temperature. The amount of malondialdehyde (MDA) generated by unsaturated fatty acids during the oxidation of a lipid system is measured by TBA [31]. The *t*-test performed for the recorded values showed that compared to the initial day, when a normal distribution could be observed, on the other days this characteristic disappeared (*p* < 0.05). Significant differences (*p* < 0.05) were registered between the TBA values of the blank sample (HAO) and the samples supplemented with BHT and OBO (100, 200, 300 and 500 ppm respectively) after performing the t-test for each test period. To support this finding, we conducted a variance analysis between samples using an ANOVA test, which yielded the same statistical result (*p* < 0.05). We also looked at possible differences that can be measured between the TBA values of OBO (100, 200, 300 and 500 ppm) and BHT. From the analysis of experimental data it can be conclude that OBO has a protective effect against the lipid oxidation of HAO, this protection increasing with the increase in the dose of OBO used. Significant protection against oxidation can be observed from the dose of 200 ppm OBO added in HAO (between 2.460 ± 0.061 µg MDA/g in day 5 and 27.107 ± 0.329 µg MDA/g in day 30) throughout the 30 days during which the analysis was performed. As the concentration of OBO added in HAO is higher, the degree of oxidative stability also increases, which suggests that the use of OBO at a concentration above 200 ppm may provide oxidative stability comparable to a concentration of 200 ppm BHT in HAO (between 2.373 ± 0.168 µg MDA/g in day 5 and 24.517 ± 0.411 µg MDA/g in day 30). 

### 2.3. EpiDerm Models

**Figure 1:**
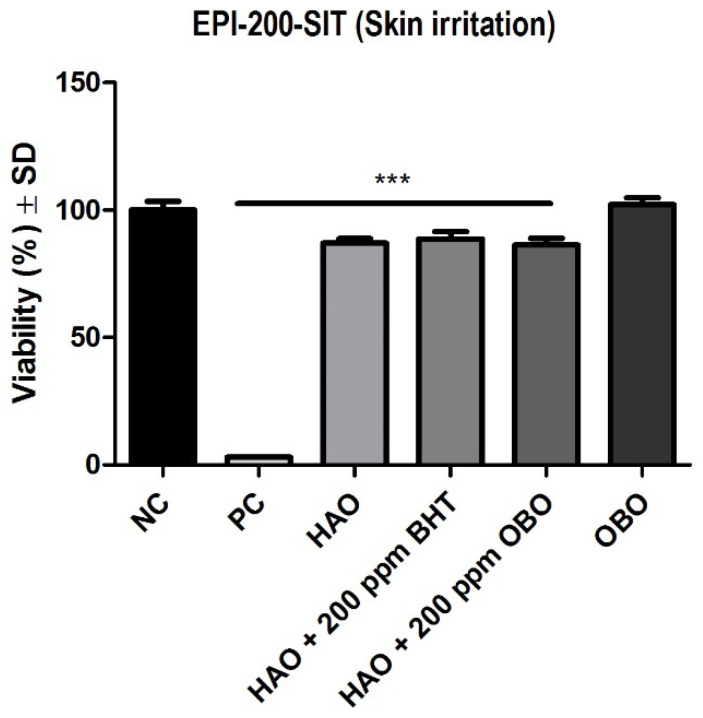
Viability of EpiDerm Skin Irritation Model (EPI-200 SIT) following application of the testes samples. One-way ANOVA followed by Dunnett’s multiple comparison test (*** *p* < 0.001) were applied. Statistical processing was performed using GraphPad Prism 5 software (San Diego, CA, USA). Negative control (NC) is represented by Dulbecco’s phosphate buffered saline (DPBS); positive control (PC) is represented by sodium dodecyl sulphate (SDS) 1%.

In Figure 1 is represented the effect of the tested oils on the EpiDerm Skin irritation model. It can be observed that the tested samples did not reduced the viability, indicating a good tolerability and that the samples do not have an irritative effect on the tissues. 

The OECD Test Guideline 439 indicates that if the viability of the sample-treated insert is under 50%, the sample is considered to be irritant [32]. All the tested combinations had a viability higher than 50%, showing that they do not have an irritant effect.

**Figure 2:**
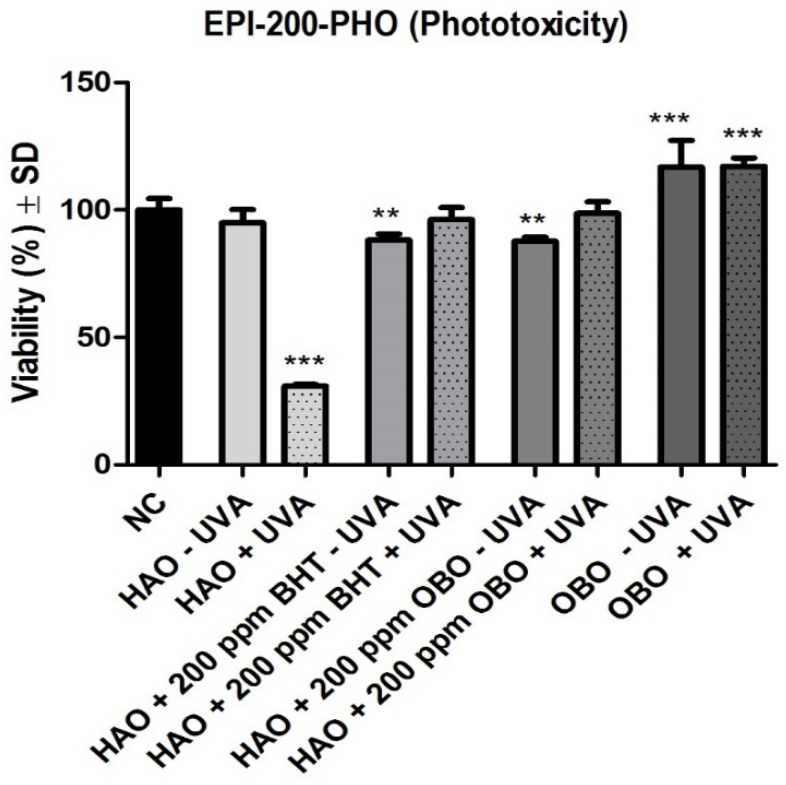
Viability of EpiDerm Phototoxicity Model (EPI-200-PHO) following application of the testes samples ± UVA exposure. One-way ANOVA followed by Dunnett’s multiple comparison test (** *p* < 0.01; *** *p* < 0.001) were applied. Statistical processing was performed using GraphPad Prism 5 software (San Diego, CA, USA). NC is represented by sesame oil.

Figure 2 depicts the effect of the tested samples on EpiDerm Phototoxicity model with or without UVA exposure. OBO and HAO + 200 ppm OBO did not reduced the viability of the tissue after UVA exposure; moreover, an increase in the viability was noticed (OBO + UVA − 117.1% viability; HAO + 200 ppm OBO + UVA − 98.7% viability). A similar effect was recorded for HAO + 200 ppm BHT after UVA exposure, with a viability of 96.1%. On the other hand the sample HAO had a phototoxic effect, reducing the viability following exposure to UVA radiation (30.9% viability). 

### 2.4. Histopathological Assessment of EpiDerm Skin Irritation Models with the Tested Oil Samples

**Figure 3:**
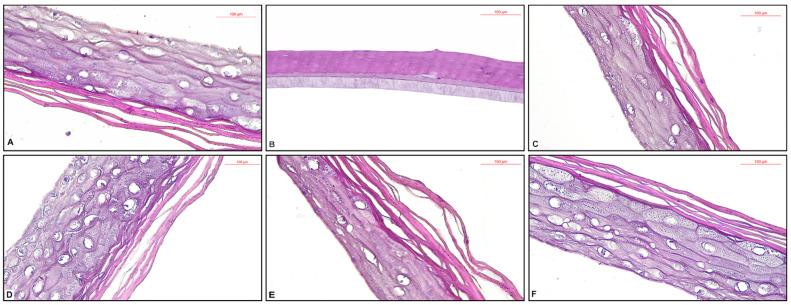
Histological aspects of EpiDerm Skin Irritation Model with the tested oil samples, hematoxylin-eosin (HE) staining: (**A**) NC group—normal histological structure, magnification ×40; (**B**) PC specimen—extensive necrosis, magnification ×40; (**C**) HAO specimen—discreet parakeratosis, magnification ×40; (**D**) HAO + 200 ppm BHT specimen—slight thickening of the stratum corneum, magnification ×40; (**E**) HAO + 200 ppm OBO specimen—mild parakeratosis, magnification ×40; (**F**) OBO specimen—slight increase of the granular layer cells, magnification ×40.

The specimens from the NC group had a normal histological structure (Figure 3A). The PC specimen—treated with SDS 5%, showed extensive necrosis (Figure 3B). The application of HAO induced discreet parakeratosis—characterized by the retention of nuclei in the stratum corneum (Figure 3C). The tissue specimen on which it was applied 200 ppm BHT added in HAO (HAO + 200 ppm BHT) showed only a slight thickening of the stratum corneum (Figure 3D). The application of 200 ppm OBO added in HAO (HAO + 200 ppm OBO) induced mild parakeratosis (Figure 3E). OBO treated specimen showed a slight increase of the granular layer cells—which have a protective role (Figure 3F).

### 2.5. Histopathological Assessment of EpiDerm Phototoxicity Models with the Tested Oil Samples ± UVA Treatment

**Figure 4:**
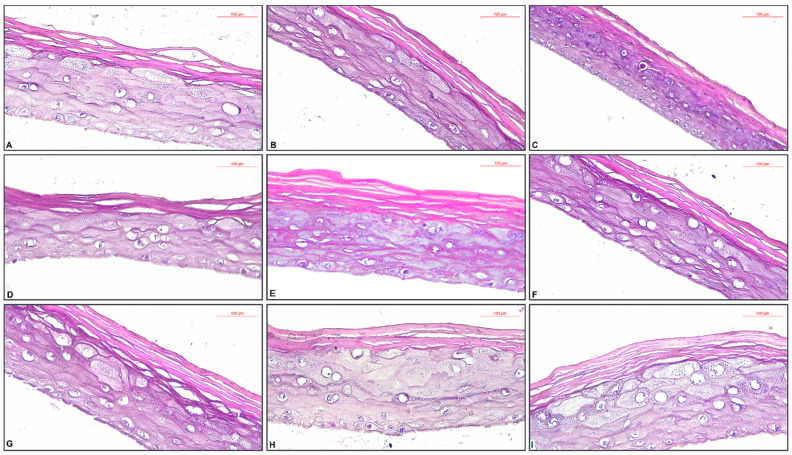
Histological aspects of EpiDerm Phototoxicity Model with the tested oil samples ± UVA treatment, HE staining: (**A**) NC group—normal histological structure, magnification ×40; (**B**) HAO − UVA specimen—slight thickening of the stratum corneum, magnification ×40; (**C**) HAO + UVA specimen—necrosis of stratum granulosum, as well as the necrobiosis of stratum spinosum, magnification ×40; (**D**) HAO + 200 ppm BHT − UVA specimen—slight thickening of the stratum corneum, magnification ×40; (**E**) HAO + 200 ppm BHT + UVA specimen—slight thickening the stratum corneum, focal increase in volume (hypertrophy) of granular cells, magnification ×40; (**F**) HAO + 200 ppm OBO − UVA specimen—discrete spongiosis, magnification ×40; (**G**) HAO + 200 ppm OBO + UVA specimen—thickening the stratum corneum, focal increase in volume (hypertrophy) of granular cells, magnification ×40; (**H**) OBO − UVA specimen—increasing in volume (hypertrophy) of granular cells, magnification ×40; (**I**) OBO + UVA specimen—thickening the stratum corneum, and the increasing in volume (hypertrophy) of granular cells, magnification ×40.

The specimens from the NC group had a normal histological structure (Figure 4A). The application of HAO but not followed by exposure to UVA (HAO − UVA) showed only a slight thickening of the stratum corneum (Figure 4B). The exposure to UVA of the specimen previously treated with HAO (HAO + UVA) determined the necrosis of the granular layer (stratum granulosum), as well as the necrobiosis of the spiny layer (stratum spinosum) (Figure 4C). The specimen treated with 200 ppm BHT added in HAO but not exposed to UVA (HAO + 200 ppm BHT − UVA) showed a slight thickening of the stratum corneum (Figure 4D). The specimen treated with 200 ppm BHT added in HAO and exposed to UVA (HAO + 200 ppm BHT + UVA) presented slight thickening the stratum corneum, focal increase in volume (hypertrophy) of granular cells (Figure 4E). The specimen treated with 200 ppm OBO added HAO but not exposed to UVA (HAO + 200 ppm OBO − UVA) showed a discrete spongiosis (Figure 4F). Exposure to UVA of the specimen previously treated with 200 ppm OBO added in HAO (HAO + 200 ppm OBO + UVA) determined the thickening of the stratum corneum, as well as focal increase in volume (hypertrophy) of granular cells (Figure 4G). The specimen treated with OBO but not exposed to UVA (OBO − UVA) showed increasing in volume (hypertrophy) of granular cells (Figure 4H). Exposure to UVA of the specimen previously treated OBO (OBO + UVA) determined the thickening the stratum corneum and the increasing in volume (hypertrophy) of granular cells (Figure 4I).

## 3. Discussion

Regarding the fatty acids content of HAO, similar results were also reported by Rosa et al. who recorded the following composition: palmitic acid 5.90%, stearic acid 3.53%, oleic acid 25.77% and linoleic acid 63.59% [33]. In another study Akkaya et al. also detailed a similar content of fatty acids for the oil obtained from several species of sunflower, namely, palmitic acid 5.0–7.6%, stearic acid 2.7–6.5%, oleic acid 14.0–39.4%, linoleic acid 48.3–74.0%, linolenic acid 0.0–0.3% [34]. On the basis of its phytochemical composition, the seed oil of sunflower has a broad range of pharmacological activities. In vitro and in vivo studies have established the dermoprotective impact of HAO as a result of increased epidermal lipid production and decreased inflammatory processes [3,35,36]. In an experiment with rats, HAO rich in oleic and linoleic acid have also displayed a cardioprotective effect, reflected by a reduction in the occurrence of life-threatening arrhythmias [37]. According to this findings, HAO may decrease total cholesterol and low-density lipoprotein (LDL) cholesterol, in addition to possessing antioxidant effects [38]. In chronic inflammatory disorders such as bronchial asthma, osteoarthritis, and rheumatoid arthritis, sunflower seeds may have a beneficial impact attributable to the anti-inflammatory effect of tocopherols. Additionally, the significant effect of vitamin E on the cardiovascular system makes sunflower seed oil advantageous for preventing atherosclerosis and, therefore, have consequences in pathologies such as coronary artery disease and stroke [39,40]. Clinical investigations have emphasized the antibacterial effectiveness of sunflower seeds in preterm newborns. Using HAO topically three times daily, improved skin health and decreased nosocomial infections contrasted to those without topical prophylaxis [41].

Timoszuk et al. (2018) also identified a similar composition in fatty acids for OBO, namley linoleic acid 73.88%, linolenic 9.24%, oleic 6.93%, palmitic 6.31%, stearic 1.88%, eicosenoic 0.55%, eicosanoic 0.31% and behenic 0.10% [29]. Balch et al. (2003) analyzed the fatty acid composition of the oil extracted from OB harvested in 1996 and 1997 and identified a palmitic acid content of 6.9% (in 1996) and 6.8% (in 1997), stearic 1.5% (in both 1996 and 1997), oleic 21.5% (in 1996) and 17.5% (in 1997), 63.2% linoleic (in 1996) and 60% (in 1997), 0.1% linolenic (in 1996) and 0.2% (in 1997) [42].

AI and IT are strictly linked to the entire fatty acid profile, better characterizing the health benefits of a plant or animal food than a simple approach based on fatty acid classes or fatty acid ratios [43]. Similar levels of SFA, MUFA and PUFA were also reported by Rosa et al., namely 10.11% SFA, 25.77% MUFA and 63.59% PUFA but also by Akkaya et al., respectively, between 7.7–14.1% SFA, 14.0–39.4% MUFA and 48.3–74.3% PUFA [33,34].

The SFA level of OBO was higher than that reported by Balch et al., respectively, at 8.59% and Montserrat-de la Paz et al. (2014), who reported a SFA content of 8.6% [20,42]. Comparing the contents of MUFA, the results obtained in the present paper for OB oil are significantly lower than the values reported by Balch et al., respectively 17.5% (1996) and 21.5% (1997) [42]. In contrast, Montserrat-de la Paz et al. described similar content for MUFA to that reported in the present paper of 8.29%. Finally, PUFA content is similar to that observed by Montserrat-de la Paz et al., respectively 83.12% and higher than that reported by Balch et al., respectively 69.5% (1996) and 68.5% (1997) [20,42].

The PUFA ω3/ω6 ratio of OB oil is comparable to that of most vegetable oils, e.g., olive oil (0.13), soybean oil (0.15) and walnut oil (0.20) [44]. It is not entirely clear what the optimal n−3/n−6 PUFA value should be for human and animal nutrition. Studies on the relationship between the ratio n−3/n−6 PUFA and the pathogenesis of many diseases indicate that the optimal ratio may vary depending on the disease or condition under consideration; this is consistent with the fact that many diseases are multigenic and multifactorial. Overall, a higher n−3/−6 PUFA ratio is more desirable in reducing the risk of many chronic diseases [45]. 

On average, the higher content of SFAs and a higher value of quality indices make their lipid fraction less suitable for animal and human nutrition [43]. 

Jianu et al. observed the same trend in TBA values when studying the effect of *Mentha × smithiana* essential oil on sunflower oil [31]. In the same line, Cocan et al. conducted a study on the effect of sweet and hot pepper seed oil on sunflower oil and observed an inhibitory effect on oxidation when using concentrations of 300 ppm chilli seeds oil, an effect comparable to the action of BHT [46]. Wang et al. also studied the effect of coriander essential oil on the oxidative stability of sunflower oil and showed that a concentration of 1200 ppm of coriander essential oil added to sunflower oil may have an inhibitory effect on the oxidation process [47].

Iqbal et al. investigated the antioxidant effect of pomegranate peel extract in the stabilisation of sunflower oil. The research team observed that concentrations of 800–850 ppm of pomegranate peel extract determined an efficient stabilisation effect compared to common additives such as BHT at the legal concentration limit [48].

The antioxidant activity of the two species was previously reported in the literature [49,50,51]. Wang et al. evaluated the antioxidant effect of the oil extracted from OB seeds and indicated that at concentrations higher than 100 µg/mL the OB oil had an increased DPPH radical scavenging activity, whereas for 2,2-azinobis(3-ethylbenzothiazoline-6-sulfonic acid) diammonium salt (ABTS) assay, the antioxidant activity was obtained at concentrations higher than 10 µg/mL [49]. In a study performed on the lipophilic fraction of OB cold-pressed oil the authors indicated a high lipid-soluble antioxidant capacity, with a value of 1.0 mM Trolox/L oil using the DPPH method [50]. Granica et al. evaluated the extract prepared from the aerial parts of OB (concentration range: 2–50 µg/mL) and showed a significant scavenging activity for hydrogen peroxide and superoxide anion assays and a moderate antioxidant activity for DPPH assay [52].

In a complex study regarding the antioxidant effect of sunflower oil at different stages during the refining process, measured by DPPH assay, the highest antioxidant activity was obtained for the crude oil, followed by the neutralized oil. The refined oil had a lower antioxidant activity and was less stable in terms of oxidation [51].

In the cells of granular layer (stratum granulosum) unfolds the terminal differentiation of keratinization. The cytoplasm of granular cells is filled with small basophilic formation which are keratohyaline granules. In the granular layer, Golgi-derived lamellar granules containing lipidic and glycolipidic substances are also found, with a reduced ovoid shape of 100 to 300 nm. After the exocytosis process, the lamellar granules create a lipidic impermeable coat around the cells. The first appearance of this layer was discovered in ancestral reptiles and determined a evolutionary transition of animals on terrestrial environment. Moreover, the keratinization process as well as the lipidic impermeable coat formation (both placed in granular cells) play a major role in the developement of skin barrier for different foreign materials [53]. Thickening of the corneum layer, as well as the increase in volume of granular cells as a result of the application of tested oils on 3D tissue models supports their protective effect. To the best of our knowledge, this is the first evaluation of this mixture on the selected 3D tissue models. 

With a different range of pharmacological functions, OB has been deployed in terms of dermatological benefits such as eczema or atopic dermatitis [54,55]. In a clinical study Chung et al. investigated the OBO properties in atopic dermatitis (AD). By evaluating the Eczema Area Severity Index (EASI) scores and also the serum fatty acids levels of 40 patients, the research group concluded that both of the doses (160 mg OBO/day, 320 mg/ twice/day) were equally effective [56]. Kim et al. investigated in vitro the extract (12.5 to 100ng/mL) using dermal fibroblasts and epithelial keratinocytes. The OB sprout extract showed significant antioxidant activity and anti-wrinkle activity which was mainly due to high levels of polyphenolic acids (gallic acid and ellagic acid) and flavonoid glycosides (luteolin 7-glucuronide and quercetin 3-glouuronide). The authors also highlighted decreased levels of MMP-1 and MMP-2, which facilitated procollagen synthesis [57].

The protective effects on the skin of the two oils, alone, or of their main active ingredients have been previously described. Danby et al. conducted a clinical study in order to determine the protective effect of HAO in AD. Therefore, the oil, rich in linoleic acid (60.9%), demonstrated the ability to maintain the stratum corneum integrity, to hydrate the skin and to reduce the severity of AD without causing erythema [3]. In the same line, in a comprehensive review, Karagounis et al. established that the topical application of sunflower oil, containing linoleic acid as main compound, improve the skin barrier function in skin diseases such as AD and xerosis [58]. Blaess et al. proposed a topical combination of linoleic acid and amitriptyline as a safe and personalized therapeutic alternative in order to treat dry and itchy skin occurred in AD, as well as the therapy with linoleic acid as a method of prophylaxis [59]. Withal, Liu et al. demonstrated that the topical application of a moisturizer formulation containing linoleic acid and ceramides alongside glucocorticoids in patients with psoriasis led to a better maintaining of the stratum corneum hydration and decreased the adverse effects of the topical application of glucocorticoids [60]. Cho et al. substantiated that ozonated sunflower oil has antimicrobial, antioxidant and protective capacities, sustaining the potential usage in cutaneous diseases [61]. HAO also reduced skin irritation induced by anionic and non-ionic surfactants in vivo, mainly due to the interaction of oil with proteins, which reduced the binding of the surfactants [62]. Freitas et al. evaluated the in vivo anti-inflammatory activity of a topical emulsion based on HAO 5%, vitamin B3 5% and glycerin 30% on Swiss mice ears. Inflammation and edema were induced by croton oil, known to be an irritant product. Thus, edema was inhibited with 49.30% and the leukocyte infiltration was suppressed [63]. In order to determine the sunscreen potential of HAO, Tamara et al. tested the effectiveness of adding different concentrations of sunflower oil to a combination of octyl methoxycinnamate and oxybenzone sunscreen formulation. Therefore, the SPF value increased directly proportional to the oil concentration (8.24% for blank; 21.26% for blank + oxybenzone 2% + octyl methoxycinnamate 5%; 26.03% HAO 1%; 26.84% for HAO 5%; 28.88% for HAO 10%) [64]. 

Because of the fact that AD is characterized by a deficiency of γ-linolenic acid, Kawamura et al. studied the effect of dietary supplementation with γ-linolenic acid in subjects with AD. Therefore, beneficial results such as decreased TEWL, reduced levels of pruritus intensity and an overall improved skin barrier function were observed [65]. Likewise, Senapati et al. pointed out that the oral treatment with 500 mg OBO improved the symptoms in patients with AD [66]. Moreover, the oral administration of 500 mg OBO twice a day for 12 weeks showed a major improvement of subjects skin parameters such as moisture (12.9%), firmness (16.7%), roughness (21.7%), fatigue resistance (14.2%), TEWL (7.7%) and elasticity (4.7%) compared to placebo group [67]. Park et al. studied the effect of OBO in preventing xerotic cheilitis as an adverse effect of isotretinoin used as treatment in patients with acne. The group treated concomitant with OBO and isotretinoin showed an improved skin water balance, compared to the control group treated with isotretinoin (TEWLlower lip = 52.18 ± 4.61, respectively 70.49 ± 3.55). Due to the content in linoleic acid, OBO demonstrated to have a moisturizing effect on the mucous membrane of the subjects. Moreover, γ-linolenic acid represents a source of anti-inflammatory eicosanoids which prevent follicular hyper keratinization [68]. Complementarily, the anti-inflammatory effect of OB hydroalcoholic extract was previously determined by our research group in vivo on an ear induced edema model. The OB extract reduced significantly the blood vessels congestion and the interstitial edema, with an overall reduction of the inflammatory infiltrate [69]. 

Furthermore, Cho et al. elaborated an antioxidant mixture of vitamin C, vitamin E, pycnogenol and OBO in order to study the in vivo anti-wrinkling capacity of the mixture on SKH-1 hairless mice exposed to UVB radiation. The research group established the fact that the orally administrated mixture inhibited the formation of matrix metalloproteinases and mitogen-activated protein kinases. Moreover, the treatment reduced the epidermal thickness and intensified the collagen synthesis. OBO seemed to act as an enhancer of the antioxidant activity of the two vitamins [70]. In addition, Koo et al. demonstrated the whitening effect of saponified OBO of UVB-induced hyperpigmentation on human skin and the anti-melanogenic activity on B16 melanoma cells, due to the increased levels of linoleic acid released by saponification [71]. Timonszuk et al. described that due to their high content of omega-6 acids (mainly linoleic acid and ***γ***-linolenic acid) OBO may influence the inflammatory diseases, including skin problems [29].

## 4. Materials and Methods

### 4.1. Preparation of Oil Samples

HAO was purchased from Solaris and OBO was purchased from Mayam. Both oils are cold-pressed vegetal oils, 100% natural, without any additives added.

### 4.2. Application of OBO to HAO

Six samples were weighed in 50 mL HAO without the use of synthetic antioxidants. 200 ppm BHT (highest legal allowable dose) was administered to one and OBO at four different concentrations was applied to four others (100 ppm, 200 ppm, 300 ppm and 500 ppm). A HAO sample devoid of antioxidants was also used as a control. For thorough homogeneity, the resulting oil mixtures were agitated using a mechanical stirrer (Heidolph, IL, USA).

### 4.3. Assessment of Fatty Acid Composition

Gas chromatography was used to assess the fatty acid profile as fatty acid methyl esters (FAME), as reported by Posta et al. [72]. To summarise, 3 mL of 20% boron trifluoride (BF_3_) in methanol (Merck KgaA, Darmstadt, Germany) was applied to a 0.1 g sample and kept for one hour in an ultrasonic water bath at 80 °C (FALC Instruments, Treviglio, Italy). Each sample was mixed with 2.5 mL sodium chloride solution 10% and 2 mL hexane and centrifuged at 3000 rpm for 15 min (Hettich, Tuttlingen, Germany). The FAMEs from the hexane fraction (1 mL) were utilised for GC-MS analysis. The fatty acid compositions were determined using a Shimadzu GCMS-QP2010 PLUS equipped with an AT-WAX column (30 m × 0.32 m) (Shimadzu, Kyoto, Japan). The carrier gas flow rate (Helium) was 1 mL·min^−1^, and the splitting ratio was 1:10. 

For 10 min, the column temperature (140 °C) was maintained, followed by a 7 °C/min gradient to 250°. The injection port temperature was 250 °C, while the ion source and GC-MS interfaces were 210 °C and 255 °C, respectively.

The NIST 05 spectrum library was used to identify fatty acids. Peak area normalisation was used to represent fatty acid composition by comparing the peak area belonging to a specific component to the total area of all peaks. All of the analyses were carried out in three different ways.

Fatty acid categories were calculated as:

SFAs = sum of C12:0 + C13:0 + C14:0 + C15:0 + C16:0 + C17:0 + C18:0 + C20:0 + C22:0 + C24:0 MUFAs = sum of C16:1 ω9 + C18:1 tr ω9 + C18:1 cis ω9 + C20:1 ω9 + C22:1 ω9 and PUFAs = sum of C18:2 ω6 + C18:2 cis ω6 + C20:4 ω3 + C18:3 ω3 + C20:5 ω3 + C20:2 ω7

The total amount of unsaturated fatty acids (UFA) was estimated as the sum of MUFA and PUFA. On the basis of the identified fatty acids, the atherogenic index (AI) and the thrombogenic index (TI) were calculated using the following Equations (1) and (2) proposed by Ulbricht and Southgate [73]:

(1) $$\mathrm{AI}=\frac{C12:0+\left(4\times C14:0\right)+C16:0}{\mathrm{PUFA}\omega 6+\mathrm{PUFA}\omega 3+\mathrm{MUFA}}$$

(2) $$\mathrm{TI}=\frac{C14:0+C16:0+C18:0}{\left(0.5\times \mathrm{MUFA}\right)+\left(0.5\times \mathrm{PUFA}\omega 6\right)+\left(3\times \mathrm{PUFA}\omega 3\right)+\left(\mathrm{PUFA}\omega 3/\mathrm{PUFA}\omega 6\right)}$$

### 4.4. Thiobarbituric Acid-Reactive Substances Assay

The TBA assay is a method frequently used for determining the degree of lipid oxidation for oil samples, based on the measurement by means of the UV spectrophotometer of the absorbance of the TBA-MDA complex at wavelength of 532–535 nm [74]. MDA is one of the intermediate oxidation products of unsaturated acids, being a dialdehyde consisting of three carbon atoms. According to the literature, MDA can be dosed following the reaction with TBA, by formation of red condensing products that are absorbed at 532–535 nm with a molar absorptivity of 27.5 absorption units/mmol [75].

The determination of oxidative activity for the samples to be analyzed by TBA method was made in accordance with the method described by Cocan et al. [46], Kikuzaki et al. [76] and Tarladgis et al. [77], with some modifications, expressed as μg of MDA per g of the sample. From each oil sample, 2 g were weighed on top of 5 mL of benzene and 4 mL of TBA (0.67%) aqueous solution (Sigma-Aldrich Chemie GmbH, Munich, Germany). Subsequently, the samples were prepared as above, were shaken with a mechanical agitator (Heidolph, Illinois, IL, USA) for 30 min and subsequently kept at rest for 10 min to separate the phase. Supernatants were taken into tubes and heated for 45 min on the water bath to 80 °C. After cooling, the absorbance of the supernatant was measured against a control sample that did not contain oil, using a UV-VIS spectrophotometer (Specord 205; Analytik Jena AG, Jena, Germany) at a wavelength of 540 nm. The calibration curve was obtained using MDA (Sigma-Aldrich Chemie GmbH, Munich, Germany) as a standard (concentration range: 9.88–49.40 μg MDA/g). 

### 4.5. EpiDerm Skin Irritation Test (OECD TG 439)

The EpiDerm model was purchased from MatTek Corporation. As a NC, DPBS was used and as PC, SDS 5%. The assay medium used in the experiments was provided by the manufacturer.

The protocol needed for OECD TG 439 was performed as indicated by the manufacturer [78]. Briefly, the inserts were removed from agarose to assay medium and incubated for 1 h. Then the inserts were transferred to fresh assay medium and incubated overnight. The second day, the tissues were dosed with 30 µL of the samples, PC or NC and incubated for 60 min. DPBS was used to wash the inserts after the exposure period, then transferred to fresh assay medium and incubated for 24 ± 2 h. On the third day, the inserts were transferred again to fresh assay medium and incubated for 18 ± 2 h. The fourth day, tissues viability was determined by means of 3-(4,5-dimethylthiazol-2-yl)-2,5-diphenyltetrazolium bromide (MTT − 1 mg/mL, MatTek MTT-100 kit). The plates were measured spectrophotometrically at 570 nm, using a microplate reader (xMark Microplate Spectrophotometer, BioRad, Japan).

The tissue viability was calculated using the following formulas [32]:

Relative viability TS (%) = [ODTS/Mean of ODNC] × 100 Relative viability NC (%) = [ODNC/Mean of ODNC] × 100 Relative viability PC (%) = [ODPC/Mean of ODNC] × 100

where: TS = test substance; NC = negative control; PC = positive control; OD = optical density.

### 4.6. EpiDerm Phototoxicity Test (OECD TG 498)

The EpiDerm model was purchased from MatTek Corporation. Sesame oil was used as control. The assay medium used in the experiments was provided by the manufacturer [79].

The protocol needed for OECD TG 498 was performed as indicated by the manufacturer. Briefly, the inserts were removed from agarose to assay medium and incubated for 1 h. Then, the inserts were transferred to fresh assay medium and incubated overnight. The second day, the tissues were dosed with 25 µL of the samples or sesame oil and incubated for 21 ± 3 h. On the third day the inserts containing the tissues were transferred from the assay medium into DPBS. The plates were exposed to UVA irradiation (+UVA plates) (covered with lids)—at 6 J/cm^2^ using the Biospectra system (Vilber Lourmat, France). The plates that were not exposed to UVA (-UVA plates) were maintained in the dark. After this step, all inserts were washed with DPBS transferred to fresh assay medium and incubated for 21 ± 3 h.

The fourth day, tissues viability was determined by means of 3-(4,5-dimethylthiazol-2-yl)-2,5-diphenyltetrazolium bromide (MTT – 1 mg/mL, MatTek MTT-100 kit). The plates were measured spectrophotometrically at 570 nm, using a microplate reader (xMark Microplate Spectrophotometer, BioRad, Japan).

The tissue viability was calculated using the following formulas [32]:

RTV (%) = [OD(sample)/OD(vehicle control)] × 100 RTV Control (%) = [OD(vehicle control)/OD(vehicle control)] × 100

where: RTV = relative tissue viability; OD = optical density.

### 4.7. Histopathological Assessment of 3D Tissue Models

A histopathological assessment of the 3D Tissue Models was performed at the end of the experiment. To preserve tissue structure and prevent degradation, the fresh specimens were immediately immersed in a fixative solution; for this 10% neutral phosphate-buffered formalin (pH 7)—which offers an adequate tissue preservation was used. After 24 h, the samples were introduced in Leica TP1020 Semi-enclosed Benchtop Tissue Processor (Leica Biosystems Nussloch GmbH, Nussloch, Germany), where the fixed tissues have been dehydrated by having extracted their water gradually immersing them through a series of increasing ethanol solutions, ending in 100% ethanol. Next, ethanol was replaced by an organic solvent miscible (toluene was used as clearing solvent) both with alcohol and the embedding medium. Then, the tissue was placed in melted paraffin (the embedding medium) at 56 °C (it evaporates the clearing solvent and promotes infiltration of the tissues with paraffin). Forwards, using the HistoCore Arcadia H + C Embedding System (Leica Biosystems Nussloch GmbH, Nussloch, Germany), the samples specimens were embedded in paraffin wax, allowing it to harden in small blocks of paraffin at room temperature. The hardened blocks with tissues and surrounding embedding medium were trimmed with Leica Biosystems RM2245 Semi-Automated Rotary Microtome (Leica Biosystems Nussloch GmbH, Nussloch, Germany) obtaining 2.5 μm thickness paraffin sections which were placed on glass slides and allowed to adhere. The samples where afterwords deparaffinized in toluene and rehydrated with ethanol in decreasing concentrations. Next, the tissue sections were stained with conventional HE stain, dehydrated with ethanol in increasing concentrations and coverslipped with Entellan^®^ New. Finally, the slides were examined by the pathologist in light microscopy using a binocular microscope Leica DM1000 LED (Leica Microsystems AG, Heerbrugg, Switzerland). Images acquisition and analysis were performed using a Leica ICC50 W Camera (Leica Microsystems AG, Heerbrugg, Switzerland).

### 4.8. Statistical Analysis

All measurements were made in triplicate, and the results were provided as mean values with standard deviation (SD). The statistical significance was marked as a value of *p* < 0.05. The statistical significance of the TBA assay was determined using a one-way ANOVA and a two-sample *t*-test with equal variances. Microsoft Excel 365 was used to process the statistical data. 

For the EpiDerm models, One-way ANOVA followed by Dunnett’s multiple comparison test (* *p* < 0.05; ** *p* < 0.01; *** *p* < 0.001) were applied. Statistical processing was performed using GraphPad Prism 5 software (San Diego, CA, USA) (www.graphpad.com, accessed on May 2022).

## 5. Conclusions

For the tested oils the highest concentration was found for the polyunsaturated acids, with linoleic acid and linolenic acid beeing representative in case of *Helianthus annuus* L. oil (HAO) and linoleic acid, arachidonic acid and linolenic beeing representative in case of *Oenothera biennis* L. oil (OBO). The higher values of PUFA/SFA, UFA/SFA and ω−6 PUFA and the lower values of SFA, TI and AI confirm the higher nutritional value of the lipid fraction in OBO compared to those in HAO. This study shows that *Oenothera biennis* L. oil may be an excellent natural choice in order to delay or prevent oxidative damage of *Helianthus annuus* L. oil. Moreover, the study asserts the protective effect of the mixture between tested oils on 3D tissue models of skin irritation and phototoxicity.

## Figures and Tables

**Table 1:** Fatty acid composition of HAO and OBO expressed as g/100 g of total fatty acids.

Shorthand Nomenclature	Type of Fatty Acid	Fatty Acids Systematic Names	Fatty Acids TRIVIAL Name	Retention Time	HAO g/100 g Total Fatty Acids	OBO g/100 g Total Fatty Acids
C12:0	-	Dodecanoic acid, methyl ester	Lauric acid	23.156	nd	0.154 ± 0.003
C13:0	-	Tridecanoic acid, methyl ester	Ginkgolic acid	27.001	nd	0.147 ± 0.004
C14:0	-	Methyl tetradecanoate	Myristic acid	30.343	nd	0.325 ± 0.007
C15:0	-	Pentadecanoic acid, 14-methyl-, methyl ester	Pentadecylic acid	35.994	nd	ω0.281 ± 0.008
C16:0	-	Hexadecanoic acid, methyl ester	Palmitic acid	36.473	7.179 ± 0.123 ^a^	6.205 ± 0.156 ^b^
C17:0	-	Heptadecanoic acid, methyl ester	Margaric acid	38.719	nd	0.033 ± 0.001
C18:0	-	Octadecanoic acid, methyl ester	Stearic acid	40.969	3.586 ± 0.110 ^a^	2.039 ± 0.057 ^b^
C20:0	-	Eicosanoic acid, methyl ester	Arachidic acid	45.004	0.138 ± 0.001 ^a^	0.275 ± 0.008 ^b^
C22:0	-	Docosanoic acid, methyl ester	Acid behenic	48.767	0.548 ± 0.003 ^a^	0.069 ± 0.002 ^b^
C24:0	-	Tetracosanoic acid, methyl ester	Lignoceric acid	52.275	nd	0.030 ± 0.001
C16:1	ω9	9-Hexadecenoic acid, methyl ester, (Z)-	Palmitoleic acid	38.014	0.158 ± 0.001 ^a^	0.094 ± 0.004 ^b^
C18:1 tr	ω9	9-Octadecenoic acid, methyl ester, €	Elaidic acid	42.054	nd	0.119 ± 0.006
C18:1 cis	ω9	9-Octadecenoic acid (Z)-, methyl ester	Oleic acid, cis	42.251	28.249 ± 1.322 ^a^	7.175 ± 0.141 ^b^
C20:1	ω9	11-Eicosenoic acid, methyl ester	11-Eicosenoic acid	46.201	nd	0.210 ± 0.007
C22:1	ω9	13-Docosenoic acid, methyl ester, (Z)-	Acid erucic	49.901	nd	0.040 ± 0.002
C18:2n6 trans	ω6	9,12-Octadecadienoic acid, methyl ester	Linoleic acid	44.045	nd	0.050 ± 0.003
C18:2n6 cis	ω6	9,12-Octadecadienoic acid (Z,Z)-, methyl ester	Linoleic acid, cis	44.324	59.941 ± 4.511 ^a^	72.093 ± 1.414 ^b^
C20:4	ω3	Methyl (Z)-5,11,14,17-eicosatetraenoate	Arachidonic acid	45.623	nd	9.812 ± 0.156
C18:3	ω3	9,12,15-Octadecatrienoic acid, methyl ester	Acid linolenic	46.371	0.208 ± 0.002 ^a^	0.233 ± 0.014 ^a^
C20:5	ω3	Methyl eicosa-5,8,11,14,17-pentaenoate	cis-5,8,11,14,17-Eicosapentaenoic acid	47.800	nd	0.033 ± 0.001
C20:2	ω7	11,13-Eicosadienoic acid, methyl ester	11,13-Eicosadienoic acid	48.034	nd	0.026 ± 0.001
					* Until 100% other compounds	* Until 100% other compounds

All measurements were made in triplicate and the values represented in the table were calculated as average values with standard deviations. The *t*-test was used to represent the significant differences between the HAO sample and the OBO. ^a,b^ the different letters in the same row indicate that the values are substantially different (*p* < 0.05).The fatty acid composition of HAO and OBO was expressed as g/100 g of total fatty acids. The sign * represents other fatty acids that add up to 100g.

**Table 2:** Fatty acid classes, fatty acid ratio and quality indices, IA and TI of HAO and OBO samples.

Sample	SFAs g/100 g Total Fatty Acids	MUFAs g/100 g Total Fatty Acids	PUFAs ω6 g/100 g Total Fatty Acids	PUFAs ω3 g/100 g Total Fatty Acids	UFA/SFA	PUFA/SFA	PUFA ω3/ ω6	AI	TI
HAO	11.451	28.407	59.941	0.208	7.733	5.252	0.003	0.087	0.240
OBO	9.49	8.196	72.143	10.078	9.530	8.664	0.140	0.085	0.188

SFAs: saturated fatty acids. MUFAs: monounsaturated fatty acids. PUFAs ω6: ω6 polyunsaturated fatty acids. PUFAs ω3: ω3 polyunsaturated fatty acids. UFA/SFA: ratio of unsaturated/saturated fatty acids. PUFA/SFA: the ratio of polyunsaturated/saturated fatty acids. ω3/ω6: ω3/ω6 ratio of polyunsaturated fatty acids. AI: Atherogenic index. TI: Thrombogenic index.

**Table 3:** The effects of OBO and BHT on TBA readings in cold-pressed HAO throughout the storage period of 30 days.

TBA (μg MDA/g)	Day 1	Day 5	Day 10	Day 15	Day 20	Day 25	Day 30
HAO	2.500 ± 0.105 ^a^	9.387 ± 0.276 ^a^	11.117 ± 0.241 ^a^	15.310 ± 0.376 ^a^	19.393 ± 0.382 ^a^	26.373 ± 0.396 ^a^	30.097 ± 0.630 ^a^
HAO + 200 ppm BHT	2.373 ± 0.168 ^a^	4.937 ± 0.195 ^b^	7.997 ± 0.175 ^b^	9.907 ± 0.570 ^b^	14.827 ± 0.403 ^b^	20.460 ± 0.901 ^b^	24.517 ± 0.411 ^b^
HAO + 100 ppm OBO	2.477 ± 0.085 ^a^	8.607 ± 0.253 ^c^	10.600 ± 0.335 ^a^	14.533 ± 0.387 ^a^	18.770 ± 0.575 ^a^	26.200 ± 0.383 ^a^	29.103 ± 0.523 ^a^
HAO + 200 ppm OBO	2.460 ± 0.061 ^a^	7.497 ± 0.313 ^d^	9.573 ± 0.182 ^c^	13.313 ± 0.409 ^c^	16.350 ± 0.342 ^c^	24.713 ± 0.421 ^c^	27.107 ± 0.329 ^c^
HAO + 300 ppm OBO	2.440 ± 0.070 ^a^	6.593 ± 0.268 ^e^	8.967 ± 0.289 ^d^	11.557 ± 0.280 ^d^	15.370 ± 0.223 ^d^	23.500 ± 0.471 ^d^	26.590 ± 0.334 ^c^
HAO + 500 ppm OBO	2.400 ± 0.195 ^a^	5.543 ± 0.214 ^b^	8.527 ± 0.278 ^b,d^	9.830 ± 0.255 ^b^	14.647 ± 0.320 ^b^	21.027 ± 0.523 ^b^	24.583 ± 0.511 ^b^

All measurements are represented as mean values with standard deviations. ^a–d^ different superscripts within the same column indicate that the data are substantially different (*p* < 0.05); the same superscripts within the same column indicate that the data are not significantly different (*p* > 0.05).

## Data Availability

The data supporting the findings of the study are available within the article.
